# Regulatory dynamic inconsistencies affecting the reluctance of Indonesia disaster budget agents to disburse expenditures

**DOI:** 10.4102/jamba.v18i1.1953

**Published:** 2026-10-09

**Authors:** Noorina Hartati, Abdul Halim, Aviandi Okta Maulana, Sumiyana Sumiyana

**Affiliations:** 1 Department of Accounting, Faculty of Economics and Business, Gadjah Mada University, Sleman, Indonesia; 2 Department of Public Financial Accounting, Faculty of Economics and Business, Universitas Terbuka, South Tangerang, Indonesia

**Keywords:** regulatory inconsistency, disaster budgeting, budget agents, expected utility, symbolic compliance, governance, accountability, collaborative networks

## Abstract

This study examined the role of Provincial or Regional Agency for Disaster (P|RAD) as budget agents in response to natural hazards and their reluctance to encourage the wise spending of disaster expenditures. This research employed qualitative critical analysis, drawing on regulatory dynamics and axiomatic framework theories. Fifteen respondents from Indonesia participated. The study highlights the Government of Indonesia’s (GoI) regulatory dynamic inconsistencies, which contribute to reluctance to spend disaster budgets. It demonstrated that budget agents engage in symbolic compliance (performativity) because of partial regulatory structures under uncertain recovery. They consequently adhere to the minimax (minimise the maximum) expected-utility axiom, but tension between compliance and fear of legal sanctions prevents disaster spending. It was found that the budget agents’ commitment is low because of regulatory complexity, sustainability needs, cost-control policies, and organisational adaptiveness. It is recommended that disaster budget agents be revitalised and restructured within regulatory ecosystems to achieve adaptability and accountability.

**Contribution:**

This article advances disaster budgeting by introducing regulatory dynamic inconsistency to explain budget agents’ reluctance to distribute funds. It contributes to behavioural public finance by applying axiomatic frameworks to reveal how regulatory ambiguity, overlapping regimes and fear of fraud lead to absentee decision-making and agents’ performativity. In practice, the study recommends revitalising the Central GoI National Boards for Disaster and P|RAD, establishing standardised cost mechanisms, simplifying regulations and enhancing social collaboration to improve the effectiveness of disaster response.

## Introduction

Firstly, this study examined the reluctance of budget agents in the Government of Indonesia’s (GoI’s) provinces and regencies to allocate funds to natural hazard relief, even when disasters occur. It then clearly explains how these agents are agitated in their distribution of disaster expenditures. Moreover, the GoI has definitive regulations governing such expenditure, such as *Law No. 24 of 2007 on Disaster Management (Law 24/2007), Government Regulation No. 21 of 2008 (GR 21/2008)* concerning disaster management implementation, and *Government Regulation No. 22 of 2008 (GR 22/2008)* concerning funding and disaster relief management. However, most provincial and regency budget agents believe that the GoI’s regulations pose significant risks to their role because of their dynamic inconsistency (Fischer 1980; Halac & Yared 2014). Secondly, the study analysed and evaluated the reasons behind agents’ agitations. It was found that their behavioural economics (Chetty 2015; Rebonato, Kainth & Melin, 2022; Savage 2019) led them to choose not to spend disaster funds because they prefer to conduct expected-utility analysis (Dang et al. 2023; Phaup 2019) rather than risk underperformance and its consequences. Because of unclear article content, they are afraid to spend disaster expenditure budgets because they face the high probability of being indicted for fraud. From a behavioural perspective, the budget agents of GoI provinces and regencies tend to conduct themselves in a manner that is overly cautious and hesitant (Donahue & Joyce 2001), akin to the concept of symbolic compliance or performativity in hegemony theory (Sumiyana et al. 2023). In short, the study revealed that budget agents in GoI provinces and regencies engage in adverse selection, consistent with the minimum expected utility axiom, which aligns with their occupational roles.

This study considered prior research related to dynamic inconsistency. Existing studies have focused on dynamic inconsistency, including economic policy (Halac & Yared 2014); commitment mechanisms (Caliendo & Findley 2019); and psychological behaviours (Ventre et al. 2023). However, this study highlights that dynamic inconsistency is caused by government regulations. Moreover, it relates this inconsistency to behavioural agents’ requirement to spend natural hazards expenditure budgets. In short, it focuses on regulatory dynamic inconsistency, characterised by unclear article content, which contributes to the reluctance to spend disaster funds (Fahlevi, Indriani & Oktari 2019). In addition, this study analysed the phenomenon of low motivation among GoI budget agents using minimin (minimise the minimum) expected utility. It was found that most previous research focused on maximum expected utilities (Borie 2023b; Echenique 2020). In contrast, it accounts for different angulations of minimin, equivalent to budget agents’ economic choices caused by regulatory dynamic inconsistency. Therefore, minimax explains the typical choices of budget agents when they face agitated situations (Ladrón de Guevara Cortés, Tolosa & Rojo 2023; Rossmo & Summers 2022) between underperforming and fraud indictments.

This study first discusses a unique issue by explaining regulatory dynamic inconsistency, primarily caused by the regulation’s appeal to expenditure cost numbers. Moreover, what became apparent is that there is an unclear relationship between deterministic inputs and disaster expenditures (Bhatti & Phaup 2015; Cetemen, Feng & Urgun 2023; Miao, Shi & Davlasheridze, 2021; Robinson & Botzen 2025). In other words, regulations governing disaster expenditures do not dictate how budget agents exercise their discretion in allocating budget funds. In addition, the regulations do not establish standard costs and drivers, allowing agents to spend funds at their discretion to help with recovery from natural hazards (Gailmard & Patty 2019; Smith 2023). Therefore, agents choosing a strategic point of budget spending for a disaster expenditure policy can still be indicted for fraud because of the numerous different perspectives on such expenditure. Consequently, the government auditor board can accuse budget agents of fraud related to funds because of unclear regulatory provisions. Moreover, government financial supervision can expose the agents’ self-interest, which can potentially lead to fraud.

The study then further shows that the consequences of dynamic uncertainty affect the GoI’s provincial and regency budget agents, who use axiomatic frameworks to allocate natural hazard budgets. Although such frameworks support professionals in business and public policy (Borie 2023a; Frisch & Clemen 1994), budget agents are unlikely to adhere to them because they prefer the minimax utility (DeCanio, Manski & Sanstad 2022; Marcarelli 2023). Inversely, when budget agents choose their preferences through maximax decision-making (Bhatti & Phaup 2015; Phaup 2019; Robinson & Botzen 2025), they are likely to be indicted for fraud because of the regulatory subjectivity of the spending of disaster budgets. The authors of this study are of the opinion that budget agents cannot commit to the disaster regulations issued by the GoI because of dynamic inconsistencies in authorised expenditures, both technical and discretionary. Therefore, GoI regulations related to the authorisation of spending natural hazard budgets do not explicitly specify, which expenditures may be made or how the discretion to distribute disaster funds should be accounted for (Borie 2023a; Miao et al. 2021). From a behavioural perspective, the budget agents of GoI provinces and regencies avoid the risk of being indicted for fraud by constructing their budgets vaguely.

This study contributes to the field of rapid response to natural hazards. It is argued that handling natural hazards involves securing people in the field and accommodating victims’ needs for food, clothing and shelter, which require funds for procuring goods and services (Dang et al. 2023; Donahue & Joyce 2001). Subsequently, if budget agents have technical and discretionary authorisation under the regulations’ protection mechanism, they will spend disaster funds allocated in their annual fiscal plan in a dynamic, consistent manner. It was furthermore revealed that there is a need for a governmental transformation process from dynamic inconsistency to consistency (Cetemen et al. 2023; Halac & Yared 2014; Phaup 2019). Moreover, regulatory dynamic consistency refers to the definitive articles and aligned content of the GoI constitution, regulations, presidential decrees, ministerial decrees and all sub-regulations. From a cost-magnitude perspective, all GoI regulations should include disaster unit-cost rules, formulations and standards. From a managerial perspective, budget agents are authorised technically and discretionarily to spend disaster funds according to their situational preferences and upper and lower cost limit policies (Miao et al. 2021).

The study also contributes to the elimination of axiomatic frameworks when the budget agents’ choice is governed by the minimax decision-making rule, replacing them with the maximax rule. It also opines that budget agents are likely to spend budgeted disaster funds by considering the maximax expected utility (Bhatti & Phaup 2015; Robinson & Botzen 2025). From a behavioural perspective, it is explained that GoI disaster management is moving from budget agents’ adverse selection in decision-making to bonding (Borie 2023a). Consequently, budget agents build the courage to spend disaster fiscal funds, which results in quick and efficient handling of natural hazards. Moreover, it is the opinion of the authors that there are no contradictions in the GoI regulations that support high-intention budget agents in distributing disaster funds. In short, budget agents have legitimised authorisation under regulatory consistency (Miao et al. 2021; Phaup 2019) to spend such funds technically and discretionarily. As a consequence, officials responsible for budget execution and fund utilization may be exempt from future prosecution for alleged fraudulent conduct.

The GoI controls natural hazard fiscal budgets for all agents on the basis of constitutions, regulations and decrees, including those at the ministerial, provincial and regency levels. This study criticises the GoI’s disaster budgets for being subject to regulatory inconsistencies and for failing to explicitly specify how disaster funds are spent. It is highlighted that the GoI lacks standard costs and activity drivers to prevent and mitigate the impact of natural hazards. For example, Indonesia faces internal and external factors related to the Atlantic and Pacific ring fires (Kadir et al. 2023). The GoI’s lack of systematic fiscal budgets for disaster prevention and recovery leads to dynamic inconsistencies for local governments because of conflicting regulations (Gailmard & Patty 2019; Halac & Yared 2014). Therefore, the set of GoI regulations discourages provincial and regional budget agents from using fiscal budgets, even if disasters have occurred.

## Literature review

### The impact of dynamic inconsistency on budget agents’ adverse selection

Regulatory dynamic inconsistency affects provincial and regency budget agents, leading to adverse selection and shirking. This study examined whether the GoI effectively controls all budget agents with regulatory dynamic consistency, enabling them to optimise their work and allocate disaster budgets efficiently for disaster management. Moreover, the agents manage the facilitation of disaster fieldwork by consistently spending disaster budgets and aligning expenditure with regulatory requirements and expected utility (Frisch & Clemen 1994; Phaup 2019). Meanwhile, it cannot be denied that GoI regulatory dynamic inconsistency impacts budget agents’ shirking behaviour (Biolsi & Kim 2021), as they consider decision-making risks, especially those not denominated by regulatory certainty. Finally, when budget agents make decisions based on correct self-preferences (Bhatti & Phaup 2015; Frisch & Clemen 1994; Robinson & Botzen 2025), they can still be indicted for fraud because they are subject to differential priorities from the GoI’s higher authorisation and the audit board. Therefore, they face a higher risk when they spend disaster budget funds without preferences.

### Budget agents’ axiomatic framework in minimin utility

Decision theory explains how policy is developed based on the probability of achieving an organisational goal (Frisch & Clemen 1994). The theory then categorises this probability into three approaches: normative, prescriptive and descriptive. Specifically, the focus of this study is on prescriptive decision-making among Provincial or Regional Agency for Disaster (P|RAD) budget agents. For example, the chair of a P|RAD budget agents makes policy decisions under consistent regulations (Biolsi & Kim 2021). Nevertheless, the chairmen of GoI regional disaster agencies work within a regulatory dynamic framework marked by inconsistency. Consequently, they have low intentions to make decisions within explicitly complex regulations, with many ambiguities in the standard cost and activity drivers. Therefore, disaster budget agents are unlikely to decide whether to spend the disaster funds on securing and recovery, as the future value utility is blurred.

From an axiomatic perspective, the budget agents of provinces and regencies should use the prescriptive decision-making branch. However, they are situationally and conditionally unexpected-utility frameworks, which contribute to their indecisiveness (Borie 2023b; Marcarelli 2023) and their reluctance to spend disaster-budgeted funds. Consequently, this study explains that budget agents face axiomatic unexpected utility when spending disaster budgets. From a behavioural perspective, they are risk-averse, reluctant to make decisions that potentially overlap with those of other agents and may lead to fraud. Consequently, by design, they should optimise their expected utility axiom for disaster budgeted fund, but reverse it to minimise that axiom through indecisiveness (Hage 1965; Rossmo & Summers 2022; Sun 2023). Because of the dynamic inconsistency of the environment, agents rely on axiomatic frameworks, which predominantly influence their adverse behaviours, such as shirking, rather than maximising the use of disaster budgets to quickly make safe and help disaster victims recover (Fischer 1980; Smith 2023; Ventre et al. 2023). Therefore, it is important to note that there is a need to transform budget agent authorisations from shirking to bonding behaviours, thereby innovating the maximax expected utilisation of disaster budget usage.

### Expenditure disbursements in disaster budgets

There are two sources of funds for disaster budgets: the Central Government of Indonesia National Boards for Disaster (GoI of NDB) and the P|RAD *(Law 24/2007, GR 21/2008, and GR 22/2008)*. Additionally, the GoI allocates these funds to routine, discretionary spending and community fundraising (*Law 24/2007, GR 21/2008, GR 22/2008*). The focus of this study is on discretionary spending and budget agents’ behaviours in their preferred work, including community fundraising, particularly in the context of disasters. Routine spending did not lead to budget agents being indicted for fraud, as it is solely for the monthly salaries of the Regional GoI civil apparatus.

When a disaster occurs, the regional budget agents can initially disburse P|RAD funds and then ask the Central GoI to transfer NBD funds to the region (*Law 24/2007, GR 21/2008, GR 22/2008*). The regional chairman of the budget agents has to wait for the issuance of a disaster emergency status by the provincial or regional head (chairman). Furthermore, they have the authority to define damaged infrastructure and victims’ psychological needs and to determine fund beneficiaries (*Law 24/2007, GR 21/2008, GR 22/2008*). It is explained in this article that the NBD and P|RAD have not structured and systemised disaster standard costs and activity drivers for disasters that occurred. Therefore, the lack of substantive guidance in the disaster emergency status, particularly the lack of standard costs and activity drivers, reinforces decision avoidance among provincial and regional budget agents, leading them to delay the disbursement of disaster funds. Consequently, they will probably be indicted for fraud if they disburse disaster funds on the basis of preferential discretionary decision-making.

### Proposition development

This study begins with the national and regional disaster revenue and expenditure budgets, which are influenced by regulatory dynamics (Douglas & Raudla 2024; Fırtın 2024; Khmara & Touchton 2024; Martin-Sardesai et al. 2024). It is highlighted that there is a set of complex regulations for disaster prevention and response, such as *Law 24/2007, GR 21/2008, GR 22/2008*, and ministerial decrees and regulations from Finance, Internal Affairs, Social Affairs, and Public Works, all of which have content conflicts or are irrelevant. Consequently, regional disaster budget agents manage their regional revenue and expenditure budgets, which are guided by axiomatic frameworks (Geiger 2024; Maharjan, Majumdar & Pournaras, 2024; Sinervo et al. 2024a). They engage in disaster prevention and response, despite not achieving optimal expected utilities (Sinervo et al. 2024b; Warren et al. 2024; Zhou et al. 2024). It is argued that regional disaster budget agents who hold disaster prevention and response responsibilities within regulatory frameworks that exhibit dynamic inconsistencies and axiomatic frameworks remain sustainable. Indonesian society exhibits highly cooperative characteristics, as evidenced by social communities that respond effectively to disasters, with citizens ready to handle disasters and save lives. They all usually spend time, energy and funds handling disasters. When individuals and social communities respond to disasters on the front line, the regional disaster budget agents work in a professional capacity, guided by morals and ethics. Based on the prior discussion, the following research propositions (P1–P3) were developed:

**P1:** How do disaster budget agents undertake the management of disaster prevention and responses in the context of regulatory dynamic inconsistencies?

**P2:** How do disaster budget agents work within the constraints of axiomatic frameworks when expected utilities are not optimal for regional revenue and expenditure budgets?

**P3:** Why do provincial and regency disaster budget agents maintain disaster management activities under regulatory dynamic inconsistencies and the constraints of axiomatic frameworks?

## Research methods and design

### Methodological approach and respondents

A critical analysis with an interpretive approach was employed to examine budget agents’ behaviour, which is relevant to their reluctance to spend disaster funds. Evidence was also sought through a critical realism approach, incorporating factual matters and cognitive thought from the provincial and regency budget disaster agents. Moreover, informants were selected whose responsibilities and accountability (Donahue & Joyce 2001) included disaster expenditure authorisation and control of budgets, particularly budget planning lists, identification of budget beneficiaries and discretionary policy for spending expenditures. Table 1 shows details of the participants.

**TABLE 1:** Informant demography.

Code	Job position	Province or regency	Interview duration (hours:minutes:seconds)
SDE-01	Chairman	Banyumas	1:39:39
SDE-02	Deputy Chairman	Banyumas	40:27
SDE-03	Analyst	Central Java	29:17
SDE-04	Chairman	Kulon Progo	1:44:28
SDE-05	Deputy Chairman	Kulon Progo	36:02
SDE-06	Chairman	Sleman	43:24
SDE-07	Deputy Chairman	Sleman	2:06:53
SDE-08	Head of Division	Boyolali	38:21
SDE-09	Head of Division	Boyolali	48:08
SDE-10	Head of Division	Central Java	1:38:20
SDE-11	Deputy Chairman	Bantul	1:17:46
SDE-12	Head of Division	Bantul	52:06
SDE-13	Volunteer	Sleman	54:32
SDE-14	Head of Division	Sleman	51:17
SDE-15	Chairman	Karanganyar	45:29

**Total**	-	-	**15:16:15**

SDE, sample disaster executive.

### Data collection, analysis and reporting

After collection, the data were analysed using a coding scheme, with triangulation across interviews, observations and documents to strengthen the findings’ credibility. The saturation principle was taken into account, referring to the point when it was believed that the collected data were sufficient to formulate and infer the outcomes of the research dimensions (Irvine & Gaffikin 2006). In-depth, open-ended research interviews were also conducted (Effendi et al. 2024). The question design focused on (1) dynamic inconsistency (Fischer 1980): unclear (imperfect) regulations, accusations and possible indictment for fraud, and commitment (in)decisiveness; and (2) axiomatic frameworks (Rossmo & Summers 2022; Sun 2023): prescriptive decisions, axiomatic expected utility, the uncertain output of decision-making, the risks of self-preference, risk aversion and the choice between shirking or bonding behaviour.

### Ethical considerations

This study received Ethical Approval Number 44/KEPK-RSB/V/25 from the Health Research Ethics Committee, Bethesda Hospital, Yogyakarta, Indonesia. Moreover, during the data collection, written informed consent was required from all participants before the interviews. The identities of participants were anonymised using coded identifiers, and the data were securely stored to maintain confidentiality. This study specifically involved government officials working on disaster budgeting. All ethical procedures complied with research standards.

## Results

From the perspectives of regulatory dynamics and axiomatic frameworks, four discussion areas were synthesised: budget agents’ absenteeism, control through vague regulations, non-expected utility and pseudo-performance. Critical analysis was conducted to develop the themes, using the ATLAS.ti and NVivo tools. In addition, the four analysis themes addressed the three study propositions, concluding that disaster prevention and responses are limited in their sustainability.

### Disaster budget agent reluctance and work on decision-making

The interview data showed that most budget agents, working as civil servants in the regencies or provinces, performed their functions, responsibilities and tasks as custodians of activities. The respondents’ opinions were synthesised to support this theme as follows:

- Most civil servants worked as regional budget agents and were not involved in disaster budgeting at the regency or provincial level. Disaster budgets typically allocated a significant proportion of their expenditures to routine expenses rather than discretionary ones. Moreover, discretionary budget expenditures awaited authorisation from regency heads or provincial governors through their emergency disaster decisions (SDE-01, SDE-02, SDE-04, SDE-05, SDE-06, SDE-07, SDE-11, SDE-15).
- Disaster budget agents were merely the custodians of the given routine budgets and waited to release discretionary budgets, even during an acute disaster emergency status (SDE-01, SDE-02, SDE-04, SDE-05, SDE-06, SDE-07, SDE-08, SDE-09, SDE-10, SDE-11, SDE-15).

It can be inferred from the above that budget agents are responsible for routine expenditures dominating the regencies’ and provinces’ disaster budgets. Compared to other agents, a significant proportion of routine expenditures affects the disaster budget, as agents’ low-grade job functions, responsibilities and tasks are impacted. From the responsibility perspective (Fagbemi et al. 2025; Padovani et al. 2025; Sinervo et al. 2024a), NBD and P|RAD are technical cost centres responsible for issuing technical and routine measurable costs. In the case of discretionary spending, disaster budget agents are not authorised to make expenditures because they must wait for a disaster emergency status statement from their superiors (Al-Naghi, Alaghbari & Barakat, 2024; Fagbemi et al. 2025; Hosseini et al. 2024). Likewise, their involvement in the budget preparation process is almost non-existent because it is assigned to the specialised regional budget office authorities (Maharjan et al. 2024; Shen et al. 2024; Sinervo et al. 2024a). Therefore, disaster budget agents lack a strategic, discretionary policy for spending even in the event of a disaster. Finally, the lack of decision-making because of unclear functions, responsibilities or tasks impacts the behaviour of the agents, especially regarding their opportunistic choices and issues of discouragement. Their adverse selections are unavoidable because of the unclear expected utility of regional disaster budgeting and related expenditure.

### Control by vague regulations

The results also revealed that vague regulations affected most disaster budget agents. As a result, their motivation and courage to optimise their work were reduced. The respondents’ opinions were synthesised to support this theme as follows:

- Provincial or Regional Agency for Disaster was governed by complex regulations, including *Law 24/2007, GR 21/2008* and several ministerial regulations from the Ministries of Internal Affairs, Finance, National Security and Social Affairs. These regulations were likely to conflict, further indicating that the sequential disaster prevention and response operating procedures were not fully aligned. The irrelevant linkage between these regulations did not encourage disaster budget agents to work optimally in carrying out their mandates (SDE-01, SDE-02, SDE-04, SDE-05, SDE-06, SDE-07, SDE-11, SDE-12, SDE-14, SDE-15).
- Typical regulations did not allow discretionary disaster budgets to be spent when the Indonesian President, governors, or heads of local government did not issue emergency statuses (SDE-01, SDE-02, SDE-04, SDE-05, SDE-06, SDE-07, SDE-08, SDE-09, SDE-10, SDE-11, SDE-12, SDE-14, SDE-15).

Provincial or regency disaster budget agents feel burdened by the diversity of unrelated regulations. Furthermore, they perceive themselves as being at high risk, especially when spending on discretionary activities (Arapis & Chatterjee 2025; Boscá, Ferri & Rubio, 2024; Chau 2024). Such vague regulations occupy their agents’ collective cognition, making it preferable for them to wait for activity orders from their superiors rather than taking the initiative to solve problems (Al-Naghi et al. 2024; Arapis & Chatterjee 2025; Mol, Van Schie & Budding, 2025), unless they are involved in routine activities and expenditures. Our study reveals that the regulations appear fragmented and disconnected from one another. Disaster budget agents are often required to act under one regulation while also complying with other overlapping regulations, which increases their risk of being accused of fraud. Likewise, the characteristics of the regulations tend to be administrative and related to accounting (Fırtın 2024; Khmara & Touchton 2024; Martin-Sardesai et al. 2024) and must be followed in such contexts. In other words, the regulations are highly bureaucratic and hierarchical, requiring significant time and effort to comply with. Finally, it has been found that disaster budget agents’ beliefs, attitudes and behaviour reflect the fact that disaster regulations are ineffective in preventing disasters and dealing with victims.

### Structuralised by axiomatic framework failure to maximise expected-utility

The results of this study provided evidence to explain why disaster budget agents did not expect utilities from routine and discretionary budgets. They tended to make cautious budgetary decisions because their scope of functions, tasks and responsibilities within the allocated budgets is very limited, to avoid potential accusations of fraud. The respondents’ opinions were synthesised to support this theme as follows:

- Complex regulations and the need for authorisations from superiors’ emergency status decisions significantly affected disaster budget agents, who often lacked clear guidelines for optimally utilising disaster budgets. Consequently, they preferred self-preferences rather than optimising their functions, tasks and responsibilities (SDE-01, SDE-05, SDE-06, SDE-07, SDE-08, SDE-11, SDE-12, SDE-14, SDE-15).
- Disaster budget agents were functionally assigned to manage routine disaster budgets, which accounted for only 1.00% of the total Regional Revenue and Expenditure Budget, compared with 20.00% allocated to Education and Culture. Such limited allocations restricted the implementation of disaster prevention and response activities (SDE-04, SDE-05, SDE-06, SDE-07, SDE-08, SDE-09, SDE-10, SDE-11, SDE-12, SDE-14, SDE-15).
- Politically elected ministers, governors and regency heads supported disaster budget agents’ self-preferences without considering the future expected utilities of disaster budgets (SDE-01, SDE-02, SDE-04, SDE-05, SDE-06, SDE-07, SDE-08, SDE-09, SDE-10, SDE-11, SDE-12, SDE-14, SDE-15).

Disaster budget agents behave in a self-preferential manner because of regulatory frameworks that depend on the disaster’s emergency status and limited budget allocations. The SDEs can do little in terms of disaster prevention and response because the budget expenditure plans focus on technical and routine measures (Al-Naghi et al. 2024; Dutta et al. 2025; Shen et al. 2024). Although there is a small budget allocation for discretionary spending, cost standards and activity drivers do not necessarily exist (Martin-Sardesai et al. 2024; Mol et al. 2025; Warren et al. 2024). Regulations overlap, leading to budget agents holding conflicting roles and being accused of fraud (Bracco, De Benedetto & Lisciandra, 2024; Kempf et al. 2024; Seidu et al. 2022). Likewise, the very small budget allocations encourage the agents to avoid extensive functions, duties and responsibilities (Al-Naghi et al. 2024; Warren et al. 2024; Zhou et al. 2024). For example, Sleman Regency has a regional revenue and expenditure budget of IDR 3.6 trillion, while the budget allocated for disasters is only IDR 3.6 billion, with 70% of this allocated to routine expenditures, including salaries, overtime incentives and allowances. On the other hand, the budget allocation for discretionary funds is so small that even a single disaster can use it up immediately.

The results of this study revealed that politically appointed ministers, governors and regency heads influence the behaviour of disaster budget agents by shaping their self-preference (Arapis & Chatterjee 2025; Douglas & Raudla 2024). It also became evident that the region’s top executives underestimate the importance of disaster agents in undertaking prevention and response efforts (Dutta et al. 2025; Fagbemi et al. 2025; Mol et al. 2025) because of the low level of funds allocated to development budget items for building facilities and infrastructure. Compared to other agents, the small share of the allocated budget makes regional superiors reluctant to participate in managing disaster revenues and expenditures. From another strategic viewpoint, disaster budget expenditures for the region’s top executives are automatic, mechanistic procedures that do not require specific policies or expertise in decision-making.

### Pseudo performance through community networks

The results of this study furthermore indicated that disaster budget agents performed poorly in managing disaster prevention and response. They were likely to ask social communities to respond to disasters first, including the provision of time, energy and funding needs. The respondents’ opinions were synthesised to support this theme as follows:

- Disaster budget agents often conducted disaster prevention and response efforts that required the involvement of social communities. Because of the small allocated disaster expenditures and the lengthy procedures for spending regional budgets, they first requested resources and funds from social communities, which were obtained through the corporate social responsibility programmes of several private companies (SDE-01, SDE-02, SDE-04, SDE-05, SDE-06, SDE-07, SDE-08, SDE-09, SDE-10, SDE-13, SDE-14; SDE-15).
- Ministerial regulations and rules were intended for accounting and administrative control regimes, leaving disaster budget agents feeling helpless when disasters occurred. They were hesitant to utilise disaster budgets because of the lack of proper procedures, which could lead to accusations of fraud. When these regulations and rules remained unchanged, disaster budget agents worked as event organisers (SDE-04, SDE-08, SDE-09, SDE-10, SDE-13, SDE-15).
- Disaster budget agents regarded themselves as the shadow guard for disaster responses because of the circumstances and bureaucratic characteristics of government budgets. Social communities served as the front guard for field disaster responses by sacrificing their resources and funds (SDE-02, SDE-04, SDE-08, SDE-09, SDE-10, SDE-13, SDE-14, SDE-15).

Disaster budget agents have functions, authorisations, responsibilities and duties to prevent and manage disasters, but their ability to act is constrained by limited authority and institutional capacity. For example, when a disaster occurs, disaster budget agents cannot act immediately to handle the disaster because of their limited discretionary regional budget (Alexeev & Yushkov 2022; Al-Naghi et al. 2024; Kurniawan 2022); complex regulations that lead to accusations of fraud (Misener et al. 2024; Seidu et al. 2022; Warren et al. 2024); very strict regulatory characteristics, such as accounting and administrative control regimes (Fırtın 2024; Martin-Sardesai et al. 2024; Padovani et al. 2025); and the characteristics of government bureaucracy (Khmara & Touchton 2024; Kurniawan 2022; Sinervo et al. 2024a). As a result, with their limited readiness, regional disaster budget agents tend to rely on social communities as the frontline for handling disaster problems in the field (Mol et al. 2025; Sinervo et al. 2024a; Zhou et al. 2024). Local populations are not merely passive victims but act as proactive ‘zero-order responders ‘ who improvise, organise and help recovery long before official assistance arrives, reflecting the essential role of grassroots networks in disaster governance (Briones, Vachon & Glantz 2019). The results of this study revealed that social communities invest time, energy and funds in fieldwork to rescue disaster victims. On the other hand, the disaster budget agents’ lack of courage to use the regional revenue and expenditure budget leads them to raise funds from social communities to finance the needs of saving victims and affected areas. Moreover, these community funds are also recommended to be received by social communities. Disaster budget agents act in this recommended way to avoid the risk of further accusations of fraud by demonstrating symbolic compliance or performativity (Sumiyana et al. 2023). Strengthening disaster governance also requires acknowledging the capacity of local actors to drive recovery and rebuild social systems through culturally grounded, participatory approaches (Marchezini 2019).

## Discussion

The four critical analysis themes have consequences for developing motivation, commitment, habits and organisational culture in NBD and P|RAD, particularly in implementing the revenue and expenditure budget. Figure 1 shows the logical structure. The process has been ongoing for a long time and shapes beliefs, attitudes and behaviours. The pervasive content practices and their consequences were then examined.

**FIGURE 1:**
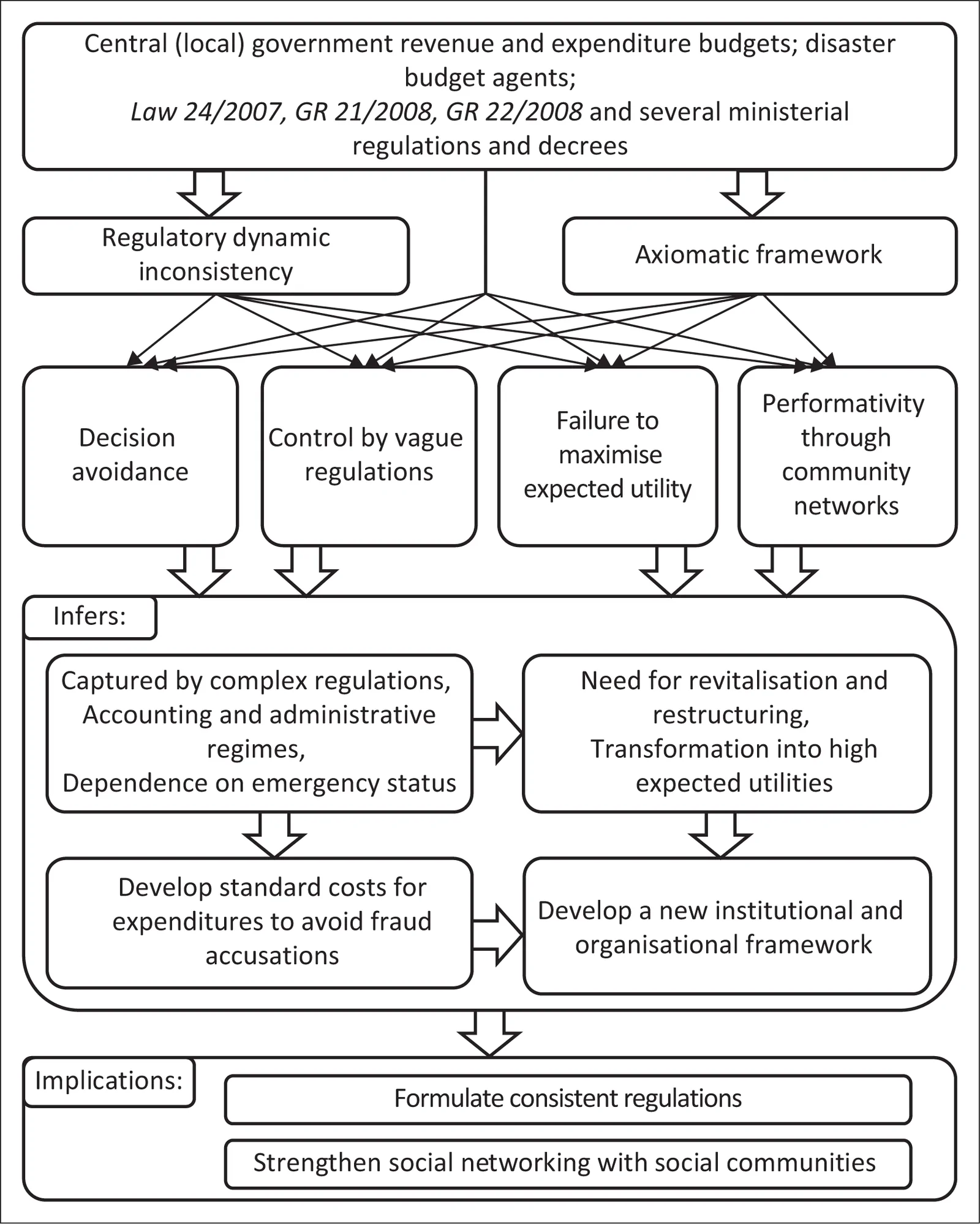
Structure of findings and implications.

As shown in Figure 1, the flow of the findings began with the contextual pressures faced by disaster budget agents, including complex regulations, small disaster budgets, dominant accounting and administrative regimes, fraud accusation risks and dependence on superior decisions for emergency status. These pressures shaped the collective mindset, motivation, commitment, habits and organisational culture of NBD and P|RAD. The figure then shows that these conditions produced low-dimensional commitment, symbolic compliance or performativity, shirking behaviour and low sustainability. Therefore, the figure leads to several implications, namely the need for institutional revitalisation and restructuring, standard cost development, regulatory consistency, budget transparency and stronger social networking to transform NBD and P|RAD into more adaptive and sustainable disaster governance institutions.

Firstly, as disaster budget agents, NBD and P|RAD run managerial and organisational systems, which are invisibly shaped by complex regulations, small disaster budgets, dominant accounting and administrative regimes, accusations of fraud, and dependence on the issuance of superior decisions for emergency status. These factors pervasively affect the collective mindset of disaster budget agents (Mol et al. 2025; Padovani et al. 2025; Seidu et al. 2022; Warren et al. 2024). Notably, they focus on low-dimensional commitments, such as disaster problem identification, internalisation and continuance. In short, the agents demonstrate symbolic job compliance or performativity (Sumiyana et al. 2023) in disaster prevention and responses because there are no further expected utilities for themselves (Al-Naghi et al. 2024; Shen et al. 2024; Sinervo et al. 2024a, 2024b). From an opportunistic behavioural perspective, they act as shirkers, which affects their performance (Bracco et al. 2024; Chau 2024; Kempf et al. 2024). The various factors in play have conditioned them, and their attitude and collective behavioural mindset cannot be divorced from the conditions and circumstances that have endured for a long time.

Secondly, the study suggests that NBD and P|RAD disaster budget agents, who can be related to institutions, budgets or funding and human resources, will face unexpected challenges in the future. This problem is synonymous with low sustainability, which should be transformed into high sustainability. It is recommended that the GoI undertake integrated reforms in institutional arrangements, budgeting systems and human resource capacity to strengthen the long-term sustainability of disaster governance (Arapis & Chatterjee 2025; Bracco et al. 2024; Douglas & Raudla 2024; Misener et al. 2024). It is thus important to note that disaster governance requires reforms in agents, budgets and human resources. In practice, this includes clarifying institutional mandates, functions and responsibilities; improving budget design and funding flexibility; strengthening staff capacity and task allocation; and establishing measurable output and outcome indicators for disaster management programmes (Khmara & Touchton 2024; Sinervo et al. 2024a, 2024b; Warren et al. 2024). From the central behaviourist perspective (Bracco et al. 2024; Chau 2024; Kempf et al. 2024), the revitalisation and restructuring of agents could change their shirking opportunistic behaviours, adverse sections and self-preferences to bonding ones because of the expected significant future utilities (Al-Naghi et al. 2024; Sinervo et al. 2024a; Zhou et al. 2024). In addition, revitalisation and restructuring could help the agents to gain typical empowerment, resulting in high sustainability.

Thirdly, the GoI, in collaboration with NBD and P|RAD, works on disaster prevention and response activities that require financial management, accounting and administrative control policies to parameterise activities and costs, taking into account government and public funds. Such parameters include state budget revenues and expenditures, primarily for disaster prevention and response activities. The security of budget expenditure practices, especially with standard costs, includes technical and discretionary activity-based costing (Kurniawan 2022; Li et al. 2024). This cost standard is crucial because it governs disaster budget agents who manage state and local government budgets and public funds, ensuring they operate without intentional or unintentional errors or irregularities. Furthermore, disaster budget agents who do not exceed the standard cost limits for disaster prevention and response expenditures are less likely to be accused of fraud (Bracco et al. 2024; Effendi et al. 2024; Seidu et al. 2022). Furthermore, the clarity of standard costs would motivate agents to utilise state budgets and public pooled funds, encouraging them to respond quickly in handling natural hazards.

Finally, standard cost is very useful for measuring and controlling the output-outcome of budget agent performance, which only leaves the completion time unmeasured and uncontrolled.

The final critical analysis concerned whether NBD and P|RAD are free from regulatory capture, have been transformed into high-sustainability organisations, operate at standard costs and employ a new institutional and organisational framework. Any new ecosystem should be disclosed to its relevant networks to ensure that NBD and P|RAD operate within a new paradigm, not just the event organisers, when a disaster occurs. Therefore, they become the first starters, ignitors and leaders (Hidajat 2024; Misener et al. 2024; Prakasa et al. 2023), controlling and managing disaster prevention and responses. National Boards for Disaster (NBD) and P|RAD can act as vanguards in solving natural hazards problems because they are rooted in a strong institutional belief, enabling their work to be conducted without constraints (Fırtın 2024; Khmara & Touchton 2024; Martin-Sardesai et al. 2024). They are free from complex regulations, axiomatic frameworks of unexpected utilities and self-preferences. Furthermore, the study reveals that the outcome is the adaptiveness of the NBDs and P|RADs as they perform their roles, functions and responsibilities dynamically. Hence, NBD and P|RAD, as agents, budgeters and human resources, achieve ultra-high sustainability in their rule and regulatory authorisations, simultaneously enhancing their mastery of disaster prevention and response.

Furthermore, it is suggested to investigate possible strategic formulations and policy innovations. Firstly, it is proposed that the complex regulations governing central and regional disaster budget agents should be reorganised to become more consistent (Edgar & Stewart 2025; Effendi et al. 2024; Ehalaiye et al. 2025; Fahlevi et al. 2019). In short, disaster regulations could be made consistent to reduce inequality, disharmony and disconnection in implementation (Edgar & Stewart 2025; Effendi et al. 2024; Ehalaiye et al. 2025; Fahlevi et al. 2019). For those in office in central and regional governments, such complex regulation leads to a high risk of being accused of fraud. In short, the laws and regulations governing national and regional hazards are issued by the state, the President, several ministries and the central or regional governments. Each level of bureaucracy issues its own regulations and rules, which do not supersede those of other levels. The necessity for a comprehensive disaster prevention and response regulation that encompasses all levels and positions within the bureaucracy is therefore emphasised (Bracco et al. 2024; Effendi et al. 2024; Ventre et al. 2023). From the perspective of asymmetrical information (Hidajat 2024; Khmara & Touchton 2024), regulation and rule control biases can lead to multiple interpretations and content distortions, further reducing the risk of fraud accusations for government officers implementing such regulations. Regulatory consistency frames government officers’ beliefs, attitudes and behaviours related to disaster budgets into a bond with greater accountability.

Secondly, P|RAD, as a disaster budget agent, should further strengthen social networking. With limited energy resources, time and funds, it establishes strong relationships with social communities (Al-Naghi et al. 2024; Sinervo et al. 2024a; Zhou et al. 2024) because of the small disaster budgets allocated. The study showed that communities are often at the forefront of responding to natural hazards. Provincial or Regional Agency for Disaster’s strengthening of social networking must also be undertaken in cooperation with state-owned, local government-owned and private firms. The reason for this is that the limited funds allocated for disaster prevention and response depend on the cost of such firms’ corporate social responsibility (Bracco et al. 2024). The results of this study revealed that the speed of material and funding expenditures from these firms exceeds that of central and regional government disaster budgets. Likewise, the firms help volunteer movements reach disaster locations directly, reflecting their high moral and ethical principles in times of disaster. Provincial or Regional Agency for Disaster, given its reliance on resources and operational speed, could strengthen social networking by building relational trust and stronger ties with communities and firms in the aftermath of disasters.

## Conclusion

This study has critically analysed NBD and P|RAD as GoI disaster budget agents, examining their low motivation to spend budgets on natural hazards through theories of regulatory dynamic inconsistencies and axiomatic frameworks. It has found behavioural evidence of the state civil apparatus functioning as disaster budget agents, engaging in decision avoidance, operating under vague regulations, without expected utilities, and engaging in symbolic compliance or performativity with social communities. It is inferred that complex regulations, numerous accounting and administrative regimes, and fears of fraud accusations hinder disaster budget agents. It is strongly recommended that NBD and P|RAD should be revitalised and structurally reorganised, supported by standard costs to allocate disaster expenditures. Therefore, NBDs and P|RADs ought to develop new institutional and organisational ecosystems by redesigning their values, missions, goals, functions, authorisations, responsibilities, main tasks and measured outcomes. Two implications are to be observed: the formulation of innovative policies to create consistent regulations and the strengthening of social networking within social communities.

The study does have limitations. It took a critical analysis position, focusing on emerging cognitive evidence. Accordingly, it opened two future research studies that discuss the comprehension of this study’s results. Firstly, future action research should detail the consistent regulations that govern and control all bureaucratic agents, including the House of Representatives, the President, and the Ministries of Finance, Internal Affairs, Social Affairs, and Public Works, as well as Provincial and Regional Local Governments. This would combine the numerous disaster regulations issued by various agencies into a single controlling regulation. Secondly, further research should advance disaster budget transparency, enabling public funds to be allocated and expended easily for disaster relief. This openness would transform the low sustainability of disaster budget agents into a higher level, particularly regarding small allocations, rigorous accounting and administrative regimes, and the shackles of expected utilities. Moreover, it examined the likelihood of inter-transfers between local governments’ disaster budgets, enabling them to support one another in disaster management.
